# Formation of pH-Responsive Cotton by the Adsorption of Methyl Orange Dye

**DOI:** 10.3390/polym15071783

**Published:** 2023-04-03

**Authors:** Mateja Kert, Jasna Skoko

**Affiliations:** Department of Textiles, Graphic Arts and Design, Faculty of Natural Sciences and Engineering, University of Ljubljana, 1000 Ljubljana, Slovenia

**Keywords:** pH sensitivity, methyl orange, conventional dyeing, cotton, colour fastness

## Abstract

The interest in pH-sensitive textile sensors is growing in the global market. Due to their low-cost production, mechanical stability, flexibility, air-permeability, washability, and reusability, they are more suitable than electronic sensor systems. The research tailored the pH-sensitive textile by applying the pH indicator methyl orange to the cotton fabric during conventional dyeing. Adsorption of methyl orange dye to cotton fabric is hindered due to electrostatic repulsive forces between dye anions and negatively charged cotton fibre. To overcome this problem, chemical modification of cotton fabric using a commercial product was performed. The pH sensitivity of the dyed fabric was spectrophotometrically evaluated. In addition, the colour fastness of dyed cotton fabric to washing, light, hot pressing and rubbing was investigated according to valid SIST EN ISO standards. The research results show that the pH-responsive cotton fabric was successfully developed. The chemical modification of cotton fabric is crucial for the increased adsorption of methyl orange dye. The halochromic effect was not only perceived spectrophotometrically but also with the naked eye. The developed halochromic cotton fabric showed poor colour fastness to light and good colour fastness to hot pressing and rubbing, while no significant improvement in colour fastness to washing was observed, even though the fabric was after-treated with a cationic fixing agent. Higher adsorption of the methyl orange dye to the cotton fabric during the dyeing process leads to less wastewater pollution after dyeing with unfixed dye and, thus, a reduction in wastewater treatment costs.

## 1. Introduction

Chromic dyes have gained more interest in the last two decades in the development of textile sensors. Their recognition in the textile industry arises from year to year, depending on market demands for passive smart textiles to which chromic dyes belong. Chromic dyes can change colour when exposed to environmental stimuli, like temperature, UV light, electric current flow, solvent, ion or pressure [1]. They are generally divided into thermochromic, photochromic, electrochromic, solvatochromic, ionochromic and piezochromic dyes. In addition, ionochromic dyes can be divided according to the ion which induces colour change into halochromic (the change of colour is caused by acid or alkali), acidochromic (the change of colour is caused by acid) and metalochromic (the change of colour is caused by metal ions) [2].

Methyl orange (MO) dye is the most commonly used pH indicator in analytical chemistry. As it can change colour from orange to red in acidic pH, it belongs to halochromic azo dye, meaning that the colour change depends on the pH value [1]. Moreover, the dye is anionic due to the sulphonic group present in the dye molecule. Even though the MO dye was not developed for the dyeing of cellulosic fibres, the studies showed that applying different indicator dyes assures the formation of pH-sensitive cellulosic textiles [3,4,5,6,7,8,9,10,11].

Halochromic dyes could be applied to textiles by conventional dyeing [3,4,5,6,7,8,9,12,13,14,15,16] and conventional printing [17,18], spraying [19], coating [17,18], sol-gel technology [5,6,7,20], during electrospinning [6,8,21,22,23] or immobilised onto the fabric channel using tetraoctylammonium bromide [24]. Among these, conventional exhaust dyeing is the most commonly used process, which does not require any change in dyeing equipment but only recommends the use of an appropriate choice of goods-to-liquor ratio (LR), the addition of chemicals, dye concentration, pH, dyeing time and temperature, and possible use of a suitable after-treatment process. The parameters mentioned above are closely related to the chemical structure of the dye and the chemical structure of the fibre, whose functional groups are responsible for the formation of different dye–fibre interactions. Moreover, the strength of dye–fibre interaction affects the wet fastness properties of dyed textiles. Already published studies show that each indicator dye requires adjustment of dyeing parameters because indicator dyes are not yet optimised for textile dyeing, thus obtaining satisfactory pH-sensitive textiles. Dye concentration, LR, the concentration of electrolyte or acid, temperature and dyeing time affect colour depth. Response time decreases with fabric density and increases with fabric hydrophilicity and its ability for quick wetting [9]. Stronger dye–fibre interactions decrease the halochromic behaviour of indicator dye. Indicator dye present in the outer region of the fibre shows a quicker response to the change of pH than the dye which penetrates the core region of the fibre [7].

Halochromic textiles provide new opportunities in the medical sector for wound dressing to monitor the healing of the wound as the pH of the skin varies during the healing process; for monitoring other human body processes, like sweat acidity, nasal discharge, etc. Another promising use of halochromic textiles due to population ageing on a global scale could be reusable diapers, where the acidity of urine could be monitored. Halochromic textiles could also be used for protecting clothing that measures pH alteration in the air in real-time, especially for those workers in the chemical industry. Environmental applications of halochromic textiles include measuring the water’s pH in textile-based filters. In the case of agrotextiles, incorporating pH-sensitive agents could give a rough estimation of the soil pH, which consequently influences crop growth [2].

Cotton, a natural cellulosic fibre, is most often used in the textile industry due to its hydrophilic character, which assures quick wetting and swelling of cotton textiles during wet textile processes. Hydroxylic functional groups on cotton fibres enable the binding of dyes and the formation of different intermolecular interactions depending on the dye structure. In aqueous solutions, cotton fibres become negatively charged, as does the methyl orange dye. The bonding between them is hindered by electrostatic repulsive forces; therefore, the addition of an electrolyte (NaCl) is strongly recommended to decrease the negative charge on the surface of cotton fibres by adsorption of sodium cations of NaCl, which consequently increase the exhaustion of the dye from the dyebath to fibres. Due to the very small molecule size of MO dye, weak intermolecular forces between dye and cotton fibres are formed, causing poor wet fastness of dyed fabric. The latter can be improved by after-treatment with a cationic fixing agent since halochromic dye’s leaching often occurs [14].

The results of studies have shown very poor exhaustion of methyl orange dye to cotton during a conventional dyeing process performed at a dye concentration of 0.5% on the mass of fibre (o.m.f.), goods-to-liquor ratio 1:50 and with an addition of 30% o.m.f. NaCl, the percentage of exhaustion was only 32% [9], which is extremely low. No studies were found to increase the adsorption of MO dye to the cotton fabric during the conventional dyeing process. Our novel approach to increase the adsorption of MO dye on cotton fabric is to chemically modify cotton fabric. However, several studies address the chemical modification of cotton by etherification, esterification, grafting and cross-linking reactions carried out by reacting with hydroxyl functional groups in cotton fibre [25]. The reaction between fibre-reactive substituted amino compounds and cotton leads to the introduction of amino groups into the cellulose fibre thus forming cationised cotton. The latter leads to high substantivity for anionic dyes due to the Coulomb attraction between the positive charge of the fibre and the negative charge of the anionic dye. Such pre-treatment of cotton improves the dye–fibre affinity and the use of salt is avoided. Among compounds used for cotton cationisation, 3-chloro-2-hydroxypropyltrimethylammonium chloride is the most researched and has reached a niche market, but poly-diallyldimethylammonium chloride combines efficacy with a cleaner process [26]. Thus, our research used a commercial product based on 1-chloro-2,3-epoxypropane, *N*-methyldiallyl amine and dimethyl diallyl ammonium chloride.

This study aims to increase the adsorption of indicator dye by replacing the addition of electrolyte (NaCl) with pre-treatment of cotton fabric with an agent that will introduce cationic groups to cotton fibres, which will result in the attraction of dye anions and consequently increase the adsorption of dye to cotton fabric. The wet fastness properties of dyed cotton will be improved by after-treatment with a cationic fixing agent. It was assumed that the cationisation of cotton fabric with a commercial product Denimcol FIX-OS would improve the adsorption of the MO dye. At the same time, the addition of electrolytes could be avoided since it is well-known that the electrolyte remains in the dyebath after dyeing because it is neither exhausted nor destroyed [27]. Moreover, the electrolyte in effluents impairs the delicate biochemistry of aquatic organisms [28]. The research also assumed that the cationisation of cotton fabric would not affect dye responsiveness. However, the change in colour due to greater depletion of the dye will be even more visually perceptible after immersing the dyed samples in buffer solutions of different pH values. Furthermore, the exhaust dyeing method was used, with a lower goods-to-liquor ratio compared to already published research [9]. The colour and the pH responsiveness of dyed fabric were spectrophotometrically evaluated using CIELAB colour space. Colour fastness to washing, light, hot pressing and rubbing was tested on dyed cotton fabrics in accordance with valid SIST EN ISO standards. 

The goal of this research is to obtain more comprehensive knowledge about the adsorption of MO dye to cationised cotton fabric and thus design textile sensors with more visually perceived colour change. The studies conducted so far have shown that azo dyes contain aromatic and azo groups in their highly toxic, carcinogenic and teratogenic molecules and are harmful to the environment and organisms to which MO dye also belongs [29]. Therefore, the higher adsorption of MO dye on cotton fabric will reduce the concentration of the dye in the wastewater after dyeing and thus contribute to a more environmentally friendly dyeing process. Moreover, in recent years, eco-friendly materials have been used to depollution textile wastewater [30]. 

## 2. Materials and Methods

### 2.1. Fabric 

In the research, chemically bleached 100% cotton fabric (CO), plain weave, mass of 114 g/m^2^, density of 48 threads/cm in warp and 32 threads/cm in weft direction, producer Tekstina d.o.o. (Ajdovščina, Slovenia), was used.

### 2.2. Dye and Auxiliaries

**Figure 1:**
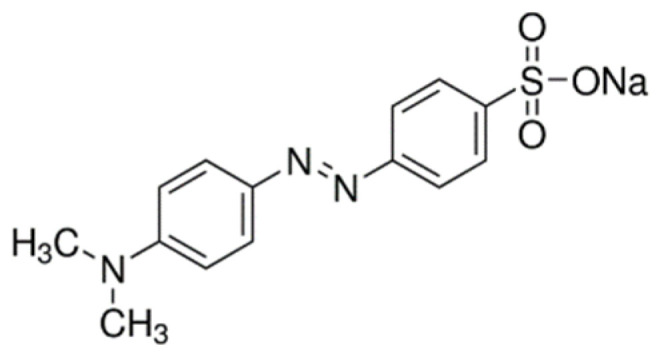
Methyl orange.

The MO dye, a pH-sensitive dye, sodium 4-{[4-(dimethylamino)phenyl]diazinyl}benzenesulfonate with a molecular weight of 327.33 g/mol (Acros Organics, Geel, Belgium) and electrolyte NaCl (Sigma-Aldrich, Oakville, Canada) were used to dye cotton fabric. The structural formula of MO is shown in Figure 1.

The reactive polyammonium compound Denimcol FIX-OS (CHT, Montlingen, Switzerland) was used for the cationisation of cotton fabric, consisting of 1-chloro-2,3-epoxypropane, *N*-methyldiallyl amine and dimethyl diallyl ammonium chloride. The cationic fixing agent—polyammonium compound Rewin MRT (CHT, Montlingen, Switzerland) was used to improve the wet fastness of the dyed cotton fabric.

### 2.3. Cationisation of Cotton Fabric

Cationisation of cotton fabric was carried out in a laboratory dyeing machine using the exhaust method. The cotton fabric was immersed in an aqueous solution of Denimcol FIX-OS, concentration of 6% o.m.f. at goods-to-liquor ratio (LR) of 1:10. After 5 min, 3 mL/L NaOH 38° Bé was added, and the fabric was treated at 50 °C for 15 min. After cationisation, the cotton fabric was rinsed twice with deionised water. Then neutralisation was carried out with 5 mL/L CH_3_COOH 30% at room temperature for 10 min. After neutralisation, the fabric was again rinsed twice with deionised water at room temperature.

### 2.4. Dyeing

**Figure 2:**
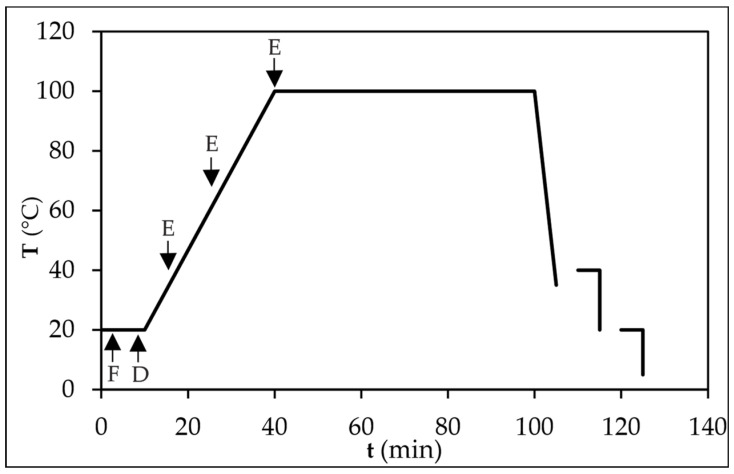
Dyeing diagram (F—fabric, D—dye solution, E—electrolyte solution).

Prior to dyeing, preliminary experiments were conducted to determine appropriate LR and the concentration of the electrolyte (NaCl), while the concentration of the MO dye was taken from literature in which the dyeing of cotton fabrics was carried out using the MO dye [8]. Thus, exhaust dyeing was carried out at LR of 1:20 and 1:40 at five different NaCl concentrations, namely 3, 10, 20, 30 and 40% o.m.f. The preliminary tests showed that the highest adsorption of the dye was obtained at lower LR and higher NaCl concentration. Therefore, the research was continued with LR of 1:20, a NaCl concentration of 30% o.m.f., and a MO concentration of 0.3% o.m.f. Dyeing of the 10 g cotton fabric was carried out in the Starlet 2 laboratory dyeing machine (Daelim, Seoul, Republic of Korea) according to the diagram shown in Figure 2. Dyeing lasted 60 min at optimum dyeing temperature (100 °C). After dyeing, two rinses were performed, the first at 40 °C and the second at room temperature with deionised water. The dyed fabric was dried in the air at room temperature (23 °C).

### 2.5. After-Treatment 

To improve the wet fastness of the dyed fabric, treatment with the cationic fixing agent Rewin MRT was carried out, using Rewin MRT at a concentration of 0.3% o.m.f. and 0.3 mL/L CH_3_COOH 80%, LR 1:20 at 40 °C for 20 min. After treatment, two rinses were performed at 40 °C and room temperature.

### 2.6. Description of the Samples

Table 1 presents the description of samples used in the research.

### 2.7. Methods of Testing

#### 2.7.1. Spectrophotometric Measurements

The calibration curve of MO was generated with the UV-vis spectrophotometer Lambda 800 (Perkin Elmer, Walton-on-the-Naze, UK). The percentage of exhaustion (*E*) was determined after dyeing cotton samples with and without cationic pre-treatment by determining the concentration of MO before and after dyeing using the calibration curve at maximum absorbance of MO (*λ_max_* = 464 nm) according to the following equation:

(1) $$E=\frac{c_{o-c_{1}}}{c_{o}}\cdot 100$$

where *E* is the percentage of exhaustion in %, *c_o_* is the concentration of MO in the dyebath at the beginning of dyeing in g/L, and *c*_1_ is the concentration of MO in the dyebath at the end in g/L.

#### 2.7.2. Colour Measurement

The colour of the dyed cotton samples was measured using a Datacolor Spectraflash 600 PLUS-CT (Lawrenceville, NJ, USA) spectrophotometer and CIELAB colour space. Measurements were made in the 400–700 nm range with a d/8° measurement geometry, under D65 illumination, 10° standard observer, with specular reflectance, included, with a 9-mm aperture. Four layers of the sample were used for the measurements. An average of ten measurements was taken for each sample. CIELAB values were determined for the chemically bleached cotton sample (CO), the dyed cotton sample (CO_D), the dyed cotton sample and treated with a cationic agent (CO_D_C), the dyed sample cationised before dyeing (CT_CO_D), and the dyed sample cationised before dyeing and then treated with a cationic fixing agent to increase wet fastness (CT_CO_D_C). In addition, the levelness of the dyed samples was calculated by taking the *K*/*S* values of 20 random points on the dyed fabric at the maximum adsorption wavelengths of the dye (*λ_max_* = 460 nm for sample CO_D and *λ_max_* = 430 nm for sample CT_CO_D) [31].

(2) $$\sigma \left(\lambda \right)=\sqrt{\frac{\sum_{i=1}^{n}{\left[\left(K/S{)}_{i,\lambda }-{\bar{(K/S}}_{\lambda }\right)\right]}^{2}}{n-1}}$$

(3) $${\bar{\left(K/S\right)}}_{\lambda }=\frac{1}{n}\sum_{i=1}^{n}{\left(K/S\right)}_{i,\lambda }$$

where *σ(λ*) is the standard deviation of individual *K*/*S* value of random points with (*K*/*S*)*_λ_*, *λ* is the wavelength of maximum dye adsorption, *n* is the number of random points tested and (*K*/*S*)*i,_λ_*, is the *K*/*S* value of each random point. The levelness increased as the *σ(λ)* value decreased. 

#### 2.7.3. Colour Fastness to Domestic and Commercial Laundering

The colour fastness of the dyed samples to domestic and commercial laundering was tested using the standard SIST EN ISO 105-C06:2012 [32]. Washing was carried out in a Gyro Wash machine (James Heal, Halifax, UK), using two different test methods, namely A1S and A1M. The detergent was a European Colourfastness Establishment (ECE) detergent without a fluorescent whitening agent. Method A1S involved washing at 40 °C for 30 min in a 150 mL washing bath containing 4 g/L ECE detergent to which 10 stainless steel balls were added. For method A1M, the washing was extended for 15 min, while the other washing conditions remained the same as for method A1S. Colour fastness to washing was visually assessed using grey scale. The staining of adjacent fabric was assessed according to the standard SIST EN 20105-A02: 1996 [33], while the colour change of the tested sample was according to the standard SIST EN 20105-A03:1996 [34].

#### 2.7.4. Colour Fastness to Light

The colour fastness of dyed samples to xenon light was tested in accordance with the standard SIST EN ISO 105-B02:2014 [35]. The samples were exposed to light in a Xenotest alpha apparatus (Atlas, Rancho Cucamonga, CA, USA) for a specified time. Light fastness was visually assessed using the blue scale. The blue scale is an 8-level scale, with level 8 corresponding to the highest light fastness and level 1 to the lowest.

#### 2.7.5. Colour Fastness to Hot Pressing

The test was conducted in accordance with the standard SIST EN ISO 105-X11:1999 [36]. The prepared sample was hot pressed at 200 °C for 15 s. The colour fastness to hot pressing was assessed by staining adjacent wet and dry materials using a grey scale in accordance with the standard SIST EN 20105-A02: 1996 [33] and the colour change of the tested sample in accordance with the standard SIST EN 20105-A03:1996 [34].

#### 2.7.6. Colour Fastness to Rubbing

The test of colour fastness of dyed fabrics against rubbing was carried out according to the standard SIST EN ISO 105-X12:2016 [37]. Dry and wet rubbing of dyed fabrics in warp and weft directions was carried out using Crockmeter M23888 (SDL ATLAS, Rock Hill, SC, USA). The colour fastness of printed fabrics to rubbing was visually assessed using a grey scale according to the standard ISO 105-A03:2019 [34].

#### 2.7.7. Determination of pH Response of Dyed Samples to Colour Change

The colour reaction of dyed fabrics to pH was determined for CT _CO_D and CT _CO_D_C samples after the test pieces were immersed in buffer solutions with different pH values, namely 2.8, 3.0, 3.2, 3.6, 4.0, 4.4, 4.8, 6.0, 7.0 and 8.0. After 5 min, all test pieces were removed from the buffer solutions, dried in air, and evaluated spectrophotometrically.

The McIlvaine buffer system was used to prepare 20 mL of buffer solutions with an appropriate amount of 0.2 M disodium hydrogen phosphate and 0.1 M citric acid, as shown in Table 2.

#### 2.7.8. Determination of Response Time

After the test piece of dyed fabric had been dipped into the buffer solution, the time (t) at which the tested fabric showed a colour change was determined.

## 3. Results and Discussion

### 3.1. Results of Spectrophotometric Measurements

The percentage of exhaustion of MO during the dyeing of cotton fabric with the addition of electrolytes was barely 8.5%. Still, it increased substantially when the dyeing of cationised cotton fabric was carried out (*E* = 99.2%). The cationisation of cotton fabric before dyeing caused a formation of the cationic charge on the fabric surface due to the presence of an ammonium group of dimethyl diallyl ammonium chloride and the amino group of *N*-methyldiallyl amine in the commercial product. A covalent bond is formed between functional hydroxyl groups of cellulose fibres and 1-chloro-2,3-epoxypropane, present in the commercial product. It is assumed that dimethyl diallyl ammonium chloride and *N*-methyldiallyl amine are bonded to 1-chloro-2,3-epoxypropane. The presence of a cationic charge on cotton led to increased adsorption of MO onto the cotton fabric. The levelness of dyeing calculated using equations 2 and 3 showed that the sample CT_CO_D is less uniformly dyed (*σ(λ)* = 1,3, *K*/*S* = 7.6, obtained at 430 nm) in contrast to the sample CO_D (*σ(λ)* = 0.08, *K*/*S* = 0.39, obtained at 460 nm). The latter could be attributed to a very narrow LR during cationisation, resulting in non-uniform adsorption of the reactive polyammonium compound to the cellulose fibres reflected in the less uniformly dyed fabric.

**Figure 3:**
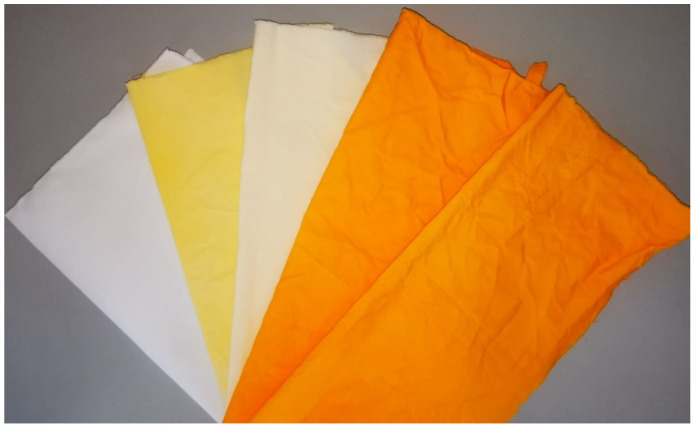
Samples before and after dyeing (from left to right: untreated, CO_D, CO_D_C, CT_CO_D and CT_CO_D_C.

The colour measurements of the dyed samples in Table 3 showed that the lightness (CIE L*) of the samples decreased after dyeing, which was expected due to the adsorption of the dye during the dyeing process. The CIE L* value decreases more for the sample CT_CO_D than for the sample CO_D. Just like the CIE L* value, the CIE a* and CIE b* values also changed. After dyeing, samples became redder (CIE a* is positive) and more yellow (CIE b* is positive) compared to the undyed sample. The cationisation of the cotton fabric before dyeing led to an additional increase of both values CIE a* and CIE b*, which made the sample redder and more yellow. The latter can be seen in Figure 3. The value of C*_ab_ increases after dyeing, which means that the samples became more saturated. A higher increase of C*_ab_ was observed in the cationised dyed sample than in the non-cationised dyed sample, which can be attributed to higher dye adsorption. The values of *h_ab_* decreased after dyeing, which was expected.

Treatment of non-cationised and cationised dyed cotton fabric with Rewin MRT after dyeing resulted in desorption of the dye MO from the cotton fabric, which was visually evident from the colour of the treatment bath and the dyed samples themselves. (Figure 3). The sample CO_D_C became lighter (the value of CIE L* increased), and the CIE a* and CIE b* values decreased compared to the CO_D sample. A slightly smaller decrease of CIELAB values after treatment with Rewin MRT was observed in the sample CT_CO_D_C compared to the sample CT_CO_D. The decrease of the values CIE a* and CIE b* is reflected in the decrease of chroma. After treatment with Rewin MRT, the dyed samples became less saturated. The reason for the greater desorption of the dye MO from the sample CO_D was attributed to the lower strength of the dye–fibre interactions compared to the sample CT_CO_D, where the surface of the cotton fabric is cationised. The dye–fibre interactions are stronger due to the electrostatic attractive forces between the cationic quaternary ammonium group of the fibre and the anionic sulphonic group of the dye. If the cellulosic fabric was not cationised before dyeing, only van der Waals forces and H-bonds could be responsible for the adsorption of the anionic dye MO to negatively charged cellulosic fibres in the dyebath. 

**Figure 4:**
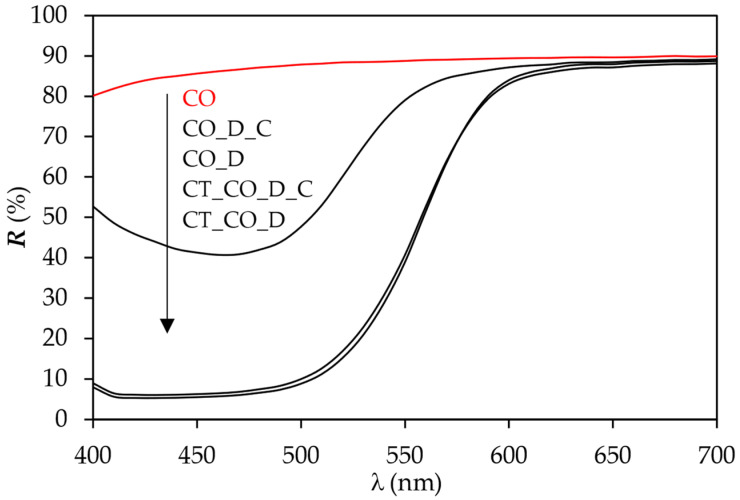
Reflection curves of samples CO, CO_D, CO_D_C, CT_CO_D and CT_CO_D_C.

**Figure 5:**
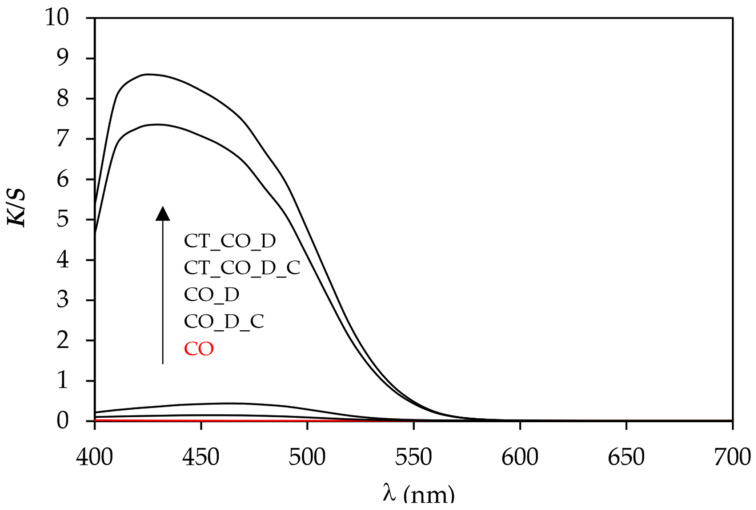
Values of *K*/*S* in dependence of the wavelength (*λ*) of samples CO, CO_D, CO_D_C, CT_CO_D and CT_CO_D_C.

The reflectance curves in Figure 4 shows that the sample CO_D reached the lowest reflectance at 460 nm and thus the highest *K*/*S* value (Figure 5), while the sample CT_CO_D reached the lowest reflectance at 430 nm and thus the highest *K*/*S* value. The latter indicated that the cationisation causes a shift of the dye adsorption to lower wavelengths, i.e., a hypsochromic shift. The sample CT_CO_D reached the highest colour depth, which is in good agreement with the percentage of dye exhaustion. After the cationic after-treatment of the samples, the R values at 430 nm or 460 nm increased, and consequently, the values of *K*/*S* decreased, which can be seen in Figure 4 and Figure 5, due to the desorption of the dye MO.

**Figure 6:**
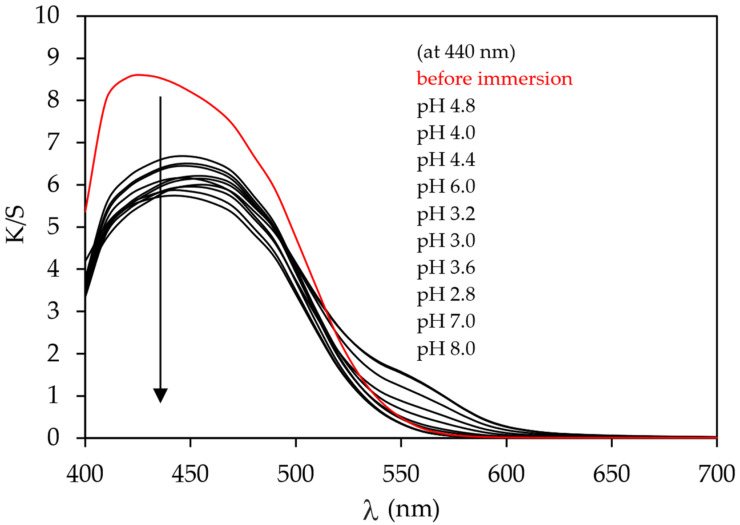
Values of *K*/*S* in dependence of the wavelength (*λ*) of sample CT_CO_D at different pH values.

**Figure 7:**
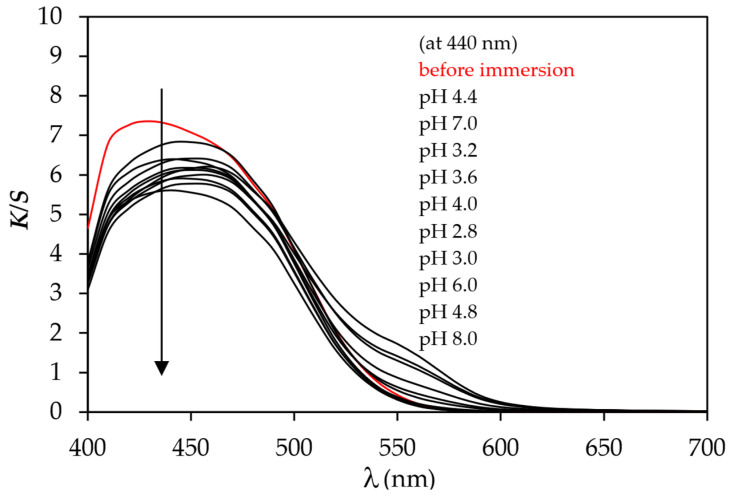
Values of *K*/*S* in dependence of the wavelength (*λ*) of sample CT_CO_D_C at different pH values.

The pH responsiveness of cotton fabric dyed in the presence of an electrolyte was not tested due to the very low adsorption of the MO dye to cotton fabric. It was tested only on dyed samples cationised before dyeing with the immersion of test pieces into buffer solutions of different pH values. Figure 6 and Figure 7 show that both samples CT_CO_D and CT_CO_D_C are pH-responsive since the bathochromic shift from 430 nm (sample before immersion) to 450 nm (sample after immersion) was noticed. The colour change was visually detected by the naked eye when the sample was wetted with buffer solution after it was immersed. Due to the very high hydrophilic character of cotton fabric, the response time was shorter than three seconds, even though another five minutes waited for complete colour change at a specific pH value of the buffer.

**Figure 8:**
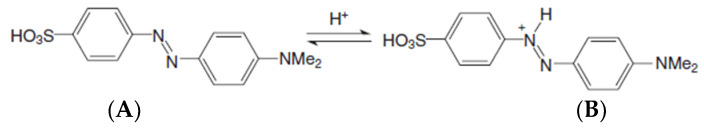
The colour change of MO dye from orange (**A**) to red (**B**) due to dye protonation.

The *K*/*S* values of samples immersed in buffer solutions decreased compared to the unsubmerged sample, which was attributed to the dye desorption from the dyed fabric. In Figure 6, the shift of curves *K*/*S* vs. *λ* was noticed to longer wavelengths or so-called bathochromic shift in buffer solutions of pH values 2.8, 3.0, 3.2, 3.6, and 4.0. The latter was ascribed to the colour change of the samples. In acidic pH, the colour of methyl orange changes from orange (A) to red (B) due to dye protonation, which is presented in Figure 8.

**Figure 9:**
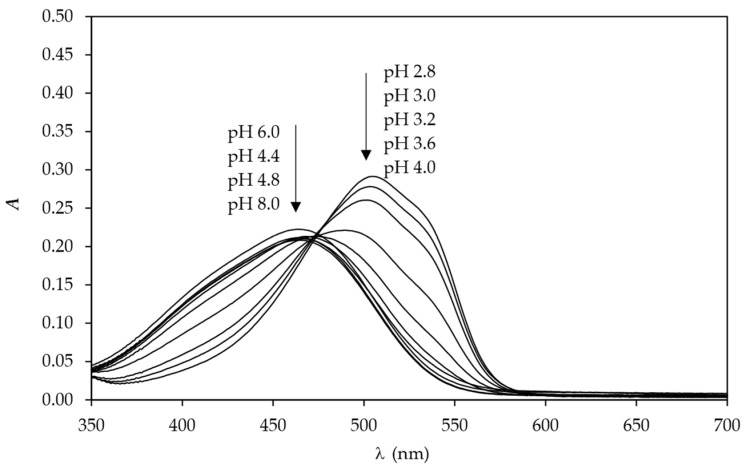
Absorption spectra of dye MO in buffer solutions of different pH values.

The CIELAB values of sample CT_CO_D, collected in Table 4, show that the lightness (CIE L*) increased with the increase of the pH value from pH 2.8 to 8.0. Values of CIE a* stay positive and almost unchanged from pH 2.8 to 3.2 but then slightly decrease in pH range from 3.6 to 8.0, whilst the values of CIE b* increase and stay positive when pH values increase from 2.8 to 8.0. This means the tested samples CT_CO_D become lighter, slightly less red and yellower by increasing pH values from 2.8 to 8.0. The same trend was noticed for sample CT_CO_D_C (Table 4). The colour change of dyed samples was detected at pH 4.0, the same pH at which the dye MO is responsive in buffer solution (Figure 9). In the solution, the shift of colour was noticed from 465 nm at pH 4.4–8.0 to 473 nm at pH 4.0 and 505 nm at pH 2.8. This means that the colour of the dye changes from orange to red at lower pH values. The bathochromic shift is clearly seen in Figure 9. Comparing the absorption spectrum of MO dye in buffer solution with curves of *K*/*S* vs. *λ* shows that the MO dye responses to pH values differ when dissolved in buffer solution than when bonded to cotton fabric. The same behaviour of dye was noticed by researcher Lien Van der Schueren et al. [5,9]. It was assumed that the cause for this phenomenon is due to dye–fibre interactions that hinder the dye from reacting faster with H+ ions in acidic buffers, as in the case of buffer solutions where the dye is in free form. 

### 3.2. Results of Colour Fastness

All samples tested have very poor wet fastness (Table 5), as grade 1 is achieved for the colour change. No staining of the first adjacent fabric was observed for CO_D, CO_D_C and CT_CO_D, while CT_CO_D_C showed stronger staining (grade 3/4). Samples CO_D and CO_D_C, as well as CT_CO_D_C, showed slight staining of the second adjacent fabric (grade 4/5), but sample CO_D_C showed stronger staining (grade 3). Therefore, washing method A1M was only performed on the sample CT_CO_D_C. The result indicates that the sample CT_CO_D_C is unsuitable for washing, as wet fastness could not be proven despite the cationic after-treatment. At the same time, it should be emphasised that the colour difference is visually less noticeable when the fabric is dyed in light (sample CO_D) than in dark (sample CT_CO_D) shades, which could be the reason for the higher visual grades obtained on adjacent fabric for samples CO_D and CO_D_C.

The results of light fastness are summarised in Table 6. They show that all the samples examined have very poor light fastness (grades 2 to 3). However, the samples CO_D and CO_C are more durable to light than the samples CT_CO_D and CT_CO_D_C. It should be mentioned that the concentration of the dye on the textile substrate may influence the grade, which is visually determined using the blue wool references. Therefore, a higher light fastness (grade 3) was determined for the samples CO_D and CO_C than for the samples CT_CO_D and CT_CO_D_C (grade 2).

Table 7 shows that the MO dye was transferred more to the wet than the adjacent dry fabric during the testing of colour fastness to hot pressing. The latter reflects lower fastness grades. However, the colour change is hardly noticeable (grades from 4 to 4/5), meaning that cotton samples dyed with the MO dye have good to very good fastness to hot pressing. For sample CT_CO_D_C, the fastness grade for the change in colour increases for half grade using a grey scale compared to sample CT_CO_D, which means that the cationic after-treatment increases colour fastness to hot pressing.

The results for rubbing fastness, compiled in Table 8, show that the tested samples are more resistant to dry rubbing than to wet rubbing. The grades for dry rubbing range from very good (grade 4/5) to excellent (grade 5), while for wet rubbing, they range from good (grade 3) to excellent (grade 5). In addition, the cationic after-treatment increases the colour fastness of both samples CO_D_C and CT_CO_D_C compared to samples CO_D and CT_CO_D.

## 4. Conclusions

Chemical modification of cotton fabric before dyeing substantially increased the adsorption of methyl orange dye to cotton during the exhaust dyeing process. As a result, dye exhaustion increased from 8.5% (uncationised cotton) to 99.2% (cationised cotton). Therefore, adding a high electrolyte concentration during the dyeing process is unnecessary. Furthermore, the colour yield is much higher, which is more acceptable from an ecological point of view, as the environment is less polluted by the discharge of the heavily coloured and saline dyebath, consequently reducing the cost of wastewater treatment.

Applying methyl orange dye to cationised cotton fabric assures the formation of halochromic textile at which the colour change from orange to red was observed at a pH of 4.0. Halochromic cotton fabric reacted immediately to the pH value, which can be attributed to the hydrophilicity of the cotton fabric. Cotton fabrics dyed with added electrolytes ensure more uniform dyeing compared to cationic pre-treated and dyed fabrics. After-treatment of the dyed samples with a cationic fixing agent does not significantly increase the wet fastness of the cotton fabric, as the colour fastness of the dyed fabric is poor when washed. The pH sensitivity of the methyl orange dye on the fabric differs from that of the dye dissolved in a buffer solution. The light fastness of the dyed cotton fabric is very poor, but the colour fastness to hot pressing and rubbing is good. Further studies will focus on improving the wet fastness of textiles dyed with methyl orange with a compound that does not affect the hydrophilicity of the cotton fabric and, thus, the pH sensitivity of the dyed textile.

## Figures and Tables

**Table 1:** Description of samples used in the research.

Sample	Description
CO	Chemically bleached cotton fabric
CO_D	Cotton fabric dyed with 0.3% o.m.f. MO and 30% o.m.f. NaCl
CO_D_CT	Cotton fabric dyed with 0.3% o.m.f. MO and 30% o.m.f. NaCl and aftertreated with 3% o.m.f. Rewin MRT and 0.3 mL/L CH_3_COOH 80%
CT_CO_D	Cationised cotton fabric and dyed with 0.3% o.m.f. MO
CT_CO_D_C	Cationised cotton fabric, dyed with 0.3% o.m.f. MO and after-treated with 3% o.m.f. Rewin MRT and 0.3 mL/L CH_3_COOH 80%

**Table 2:** Composition of the 20 mL buffer solutions used in the research.

pH	0.2 M Na_2_HPO_4_ (cm^3^)	0.1 M (C_6_H_8_O_7_) (cm^3^)
2.8	3.17	16.83
3.0	4.11	15.89
3.2	4.94	15.06
3.6	6.44	13.56
4.0	7.71	12.29
4.4	8.82	11.18
4.8	9.86	10.14
6.0	12.63	7.37
7.0	16.47	3.53
8.0	19.45	0.55

**Table 3:** Colour values CIELAB, chroma (C*_ab_) and hue angle (*h_ab_*) of undyed and dyed samples.

Sample	L*	a*	b*	C*_ab_	*h_ab_* (°)
untreated	95.41	−0.24	2.22	2.23	96.24
CO_D	88.34	7.63	29.54	30.51	75.52
CO_D_C	91.47	3.19	17.08	17.38	79.42
CT_CO_D	72.42	36.00	74.78	83.00	64.30
CT_CO_D_C	73.05	34.09	72.31	79.94	64.76

**Table 4:** Colour values CIELAB, chroma (C*_ab_) and hue angle (*h_ab_*) of samples CT_CO_D and CT-CO_D_C after immersion in buffer solutions of different pH values.

Sample	pH	L*	a*	b*	C*_ab_	*h_ab_* (°)
	2.8	58.27	33.13	43.08	54.35	52.43
	3.0	58.53	33.22	44.04	55.18	52.99
	3.2	61.00	33.29	48.54	58.86	55.56
	3.6	64.42	32.26	53.31	62.32	58.82
CT_CO_D	4.0	67.00	32.84	59.77	68.20	61.22
	4.4	70.23	32.68	65.08	72.83	63.34
	4.8	71.50	33.09	68.04	75.66	64.07
	6.0	74.50	32.21	71.26	78.20	65.68
	7.0	74.82	31.84	70.73	77.57	65.76
	8.0	74.73	31.55	69.88	76.67	65.71
	2.8	58.16	35.66	43.51	56.27	50.66
	3.0	59.76	33.79	45.60	56.75	53.45
	3.2	60.45	33.45	48.35	58.79	55.32
	3.6	64.60	32.41	54.52	63.43	59.27
CT_CO_D_C	4.0	68.01	32.36	60.08	68.25	61.68
	4.4	69.46	32.92	65.11	72.96	63.18
	4.8	73.11	31.54	67.35	74.37	64.91
	6.0	74.88	31.82	70.97	77.78	65.85
	7.0	74.43	32.37	71.96	78.91	65.78
	8.0	75.15	30.59	69.96	76.35	66.38

**Table 5:** Colour fastness to commercial and domestic washing using methods A1S and A1M* (* method A1M was performed only on sample CT_CO_D_C).

Sample	Fastness Grade Using Grey Scale		
	Staining of the First Adjacent Fabric	Staining of the Second Adjacent Fabric	Change in Colour
CO_D	5	4/5	1
CO_D_C	5	4/5	1
CT_CO_D	5	3	1
CT_CO_D_C	3/4	5	1
CT_CO_D_C	3/4 *	4/5 *	1 *

**Table 6:** Colour fastness to light.

Sample	Fastness Grade Using Blue Wool References
CO_D	3
CO_D_C	3
CT_CO_D	2
CT_CO_D_C	2

**Table 7:** Colour fastness to hot pressing.

Sample	Fastness Grade Using Grey Scale		
	Staining of Wet Adjacent Fabric	Staining of Dry Adjacent Fabric	Change in Colour
CO_D	4	4/5	4/5
CO_D_C	4/5	5	4/5
CT_CO_D	3	4	4
CT_CO_D_C	4	4/5	4/5

**Table 8:** Colour fastness to rubbing.

Sample	Fastness Grade Using Grey Scale			
	Dry Rubbing		Wet Rubbing	
	Warp Direction	Weft Direction	Warp Direction	Weft Direction
CO_D	5	5	4/5	4/5
CO_D_C	5	5	5	5
CT_CO_D	4/5	4/5	3	3
CT_CO_D_C	4/5	4/5	4	3/4

## Data Availability

The data presented in this study are available on request from the corresponding author.
